# Enhancing the Promise of Drug Repositioning through Genetics

**DOI:** 10.3389/fphar.2017.00896

**Published:** 2017-12-06

**Authors:** Jayne-Louise E. Pritchard, Tracy A. O’Mara, Dylan M. Glubb

**Affiliations:** Department of Genetics and Computational Biology, Molecular Cancer Epidemiology, QIMR Berghofer Medical Research Institute, Brisbane, QLD, Australia

**Keywords:** drug repositioning, genetics, therapeutics, GWAS, eQTL, functional genomics

## Abstract

The development of new drugs has become challenging as the necessary investments in time and money have increased while drug approval rates have decreased. A potential solution to this problem is drug repositioning which aims to use existing drugs to treat conditions for which they were not originally intended. One approach that may enhance the likelihood of success is to reposition drugs against a target that has a genetic basis. The multitude of genome-wide association studies (GWASs) conducted in recent years represents a large potential pool of novel targets for drug repositioning. Although trait-associated variants identified from GWAS still need to be causally linked to a target gene, recently developed functional genomic techniques, databases, and workflows are helping to remove this bottleneck. The pre-clinical validation of repositioning against these targets also needs to be carefully performed to ensure that findings are not confounded by off-target effects or limitations of the techniques used. Nevertheless, the approaches described in this review have the potential to provide a faster, cheaper and more certain route to clinical approval.

## Introduction

Over 6,000 human medical conditions have defined molecular phenotypes (Johns Hopkins University, 2017) but only ∼500 conditions have approved therapies (National Institutes of Health, 2015). Furthermore, many approved therapies have suboptimal efficacy or are accompanied by unacceptable toxicity. Despite scientific advancements, drug development remains challenging as development time and costs are increasing while drug success rates are low. Indeed, for every US dollar spent on research and development, the number of new drugs that are approved by the US Food and Drug Administration (FDA) has roughly halved every 9 years since 1950 (Scannell et al., 2012). The magnitude and duration of this phenomenon suggest that current approaches addressing the research and development productivity problem are having a weak effect. Not surprisingly, pharmaceutical companies often cannot afford to pursue development of promising drug candidates. It is apparent that alternative directions are required to address these critical issues. This review will focus on promising approaches to improve the success of therapeutic development by repositioning existing drugs against molecular targets identified from genetic studies.

## Drug Repositioning

Drug repositioning, also known as drug repurposing, aims to use existing therapies or drugs that have stalled in development to treat conditions for which they were not originally intended. Given that in the US alone there are ∼3000 approved drugs (U.S. Department of Health and Human Services, 2017) and thousands more which have not reached clinical approval, drug repositioning supplies a vast armamentarium to expedite the development of new therapies. The development of a new drug takes on average 13–15 years and costs between US$2–3 billion (Nosengo, 2016) with only a ∼10% chance that a new therapy will be successfully approved by government regulatory agencies (Smietana et al., 2016). In contrast, drug repositioning represents increased efficiency and lower costs because candidates already have established safety profiles from Phase I clinical trials, with time to approval estimated at 6.5 years at an average cost of US$300 million (Nosengo, 2016). One of the most successful drug repositioning examples is thalidomide, a drug whose use was originally discontinued due to severe skeletal birth defects (Kim and Scialli, 2011). After repositioning, thalidomide and its derivatives are now indicated for the treatment of multiple myeloma and a skin condition related to leprosy with sales revenues of billions of dollars.

## Identifying Drug Targets From Genetic Studies

An approach that may increase the likelihood of drug repositioning success is the use of genetic studies to identify “druggable” targets. Drugs that have been linked to disease traits through genetic studies are reported to be twice as likely to be clinically approved compared to drugs with no such links (Nelson et al., 2015). The advent of large-scale genetic studies, primarily involving genome-wide association studies (GWASs), has greatly advanced our knowledge of the genetic basis for many diseases (Visscher et al., 2017), allowing researchers to leverage this information to identify targets for therapy. Indeed, genetic studies have identified a large number of genes whose proteins are already targeted by drugs used in clinical practice. For example, the genes encoding drug targets of tamoxifen (*ESR1*) and aromatase inhibitors (*CYP19A1*) have been linked to genetic variation associated with risk for breast (Dunning et al., 2016) and endometrial cancer (Thompson et al., 2016), diseases that are treated using these drugs. Moreover, genetic studies are revealing novel drug targets such as *PCSK9*. It was initially reported that *PCSK9* nonsense mutation carriers had lower plasma levels of LDL cholesterol and a significantly reduced risk of coronary heart disease (Horton et al., 2007). A common genetic variant (rs11206510) ∼10 kb upstream of *PCSK9* was also subsequently found to associate with coronary heart disease (Schunkert et al., 2011). Based on these genetic findings, two human monoclonal antibodies have been developed to lower cholesterol by inhibiting PCSK9 (Markham, 2015; Paton, 2016) and one of these drugs was recently found to lower LDL cholesterol levels by ∼60% in a large clinical trial (Sabatine et al., 2017). Additionally, findings from genetic studies have led to drug repositioning as is demonstrated by secukinumab, an antibody therapy that targets IL-17A, which was originally tested for efficacy in the treatment of psoriasis, rheumatoid arthritis and uveitis (Hueber et al., 2010). However, IL-17A belongs to an immune axis with IL-23 (Gaffen et al., 2014) and the association of a variant (rs11209032) ∼15 kb downstream of the gene encoding the IL-23 receptor (*IL23R*) with ankylosing spondylitis (Burton et al., 2007) thus provided a rationale for repositioning secukinumab to treat this additional inflammatory disease.

## Genome-Wide Association Studies (GWASS)

Although GWAS have transformed the study of common genetic variation over the last 10 years, there has been criticism of their limited clinical impact. However, sample sizes for many diseases have only recently reached sufficient size to detect significant numbers of genome-wide significant loci (Visscher et al., 2017). As of November 2017, the GWAS catalog contains ∼53,000 unique variant-trait associations for more than 800 human traits and diseases (MacArthur et al., 2017), likely representing a large number of genes that could provide targets for drug repositioning studies. While ∼10% of GWAS variants affect the coding sequence and, therefore, have a high probability of affecting the function of the gene or encoded protein in which they are located, the vast majority of GWAS variants are found in intergenic or intronic regions and their gene targets are less clear. These non-coding variants likely affect the trait of interest through regulation of gene expression, but determining their gene targets is a complex task because GWAS variants may only regulate the nearest gene one third of the time (Gusev et al., 2016; Zhu et al., 2016). Long-range chromatin looping interactions allow genetic variants to potentially regulate a large number of genes over megabase distances (Mifsud et al., 2015). Thus, assigning the gene nearest a GWAS variant as a target may lead to false assignment of causation. An example of this is studies that were conducted on *FTO*. Intronic variants in *FTO* had been associated with obesity and body mass index and *FTO* was thought to be the regulatory target of these variants (Dina et al., 2007; Frayling et al., 2007). However, it was later determined through functional genetic experiments and mouse knockout studies that *IRX3*, a gene distally located from the GWAS variants, was the likely causal gene (Ragvin et al., 2010; Smemo et al., 2014).

The complexity of determining the causal genes through which trait-association variants act has constituted a major roadblock in the clinical translation of GWAS findings. However, in recent years, workflows have been developed to establish these causal genes (Edwards et al., 2013) and much progress is being made toward systematically identifying these genes using new functional genomic techniques that assess chromatin interactions and gene expression associations.

## Approaches to Identify the Target Genes of Trait-Associated Variants

Sophisticated computational approaches have been developed to identify disease-gene associations from GWAS data and include NetWAS which incorporates functional genomic data to identify tissue-specific gene networks (Greene et al., 2015). However, these bioinformatic tools require firstly assigning trait-associated variants to a gene. Experimental techniques such as chromatin Confirmation Capture (3C) and related high-throughput techniques (e.g., 5C, ChIA-PET, Hi-C) have been successful in identifying long-range chromatin interactions between genomic regions (Schmitt et al., 2016). These data are extremely useful in identifying interactions between GWAS loci and potential target genes (Michailidou et al., 2015; Mifsud et al., 2015; Cheng et al., 2016). However, large scale chromatin interaction assays which assess all possible interactions (e.g., Hi-C) are costly, with billions of sequencing reads required to ensure suitable resolution and confidence to accurately assess interactions. Fortunately, there are numerous databases housing publicly available data from chromatin confirmation experiments across multiple tissues and cell types (**Table 1**). These databases also provide for standardization of the complex analysis of such experiments. 3C also allows for the identification of allele-specific interactions between genes and genomic regions containing trait-associated alleles (Glubb et al., 2017), implicating the involvement of the trait-associated variant in the chromatin interaction. Importantly, bioinformatic tools have now been developed to systematically identify allele-specific interactions from large-scale 3C experiments (Servant et al., 2015; Li et al., 2017). Approaches have also been developed to integrate functional genomic data to predict interactions between genes and regulatory regions (**Table 1**; e.g., PreSTIGE and IM-PET). These approaches take advantage of the vast amount of public data provided by consortia such as ENCODE (Encode Project Consortium, 2012) and Roadmap Epigenomics Project (Chadwick, 2012), as well as data made publicly available by researchers, such as the Gene Expression Omnibus (Barrett et al., 2013). Moreover, the experiments required to generate data for these integrative approaches, commonly ChIP-seq and RNA-seq, can be performed at a fraction of the cost of a Hi-C experiment.

**Table 1:** Databases and tools for the identification of gene targets.

Database/tool type	Name	Description	Website	Citation
eQTL	ExSNP	The ExSNP database can be used to query genetic variants and genes for associations with gene expression using 16 publicly available human eQTL studies. These data allow tissue- and population-specific eQTLs to be identified.	www.exsnp.org/	Yu et al., 2016
	Blood eQTL browser	The Westra blood dataset is an eQTL meta-analysis of peripheral blood samples from ∼5,300 individuals with replication in ∼2,700 individuals. *Cis*- and *trans*-eQTLs can be queried by gene or variant.		Westra et al., 2013
	DAGS	Depression Genes and Network results of eQTL analyses for blood samples from 922 individuals are available for download.	http://dags.stanford.edu/dgn/	Battle et al., 2014
	GTEx	GTEx has matching gene expression and genotype information for >50 human tissues from >400 individuals. eQTL and splice QTL data can be queried by gene or variant.	https://gtexportal.org/home/	Ardlie et al., 2015
	Braineac	The Braineac dataset contains 134 neurodegenerative-free brains and can be queried for variants and genes associated with neurological disorders. Up to 12 brain regions were extracted per brain in parallel for mRNA quantification. Results of eQTL and genotyping analyses can be downloaded.	www.braineac.org/	Ramasamy et al., 2014
Functional genomic	NCBI GEO	NCBI GEO database contains 4348 genomic data sets and 2,184,488 samples that are cross-linked from high-throughput microarray and next-generational sequence functional genomic data sets. It can be queried for raw, processed or meta-data. All data are also available for download.	www.ncbi.nlm.nih.gov/geo/	Barrett et al., 2013
	ENCODE	ENCODE contains data from 13,393 biosamples. Queries can be made for the following experimental data: open chromatin (DNase-seq, ATAC-seq); histone mark enrichment (ChIP-seq); transcription factor binding (TF ChIP-seq); gene expression (RNA-seq); and 3D chromatin interactions (ChIA-PET).	www.encodeproject.org/	Encode Project Consortium (2012)
	Roadmap Epigenomics Project	The Roadmap Epigenomics Project contains includes 1,821 histone modification datasets, 360 DNase datasets, 277 DNA methylation datasets, and 166 RNA-Seq datasets. Epigenomics data can be browsed and downloaded.	www.roadmapepigenomics.org/	Roadmap Epigenomics Consortium et al., 2015
	GWAS3D	Sets of genetic variants can be queried to identify the most probable functional variants affecting transcriptional regulation, prioritize leading variants, evaluate deleteriousness of genetic variants affecting the gene regulation and annotate genetic variants from a regulatory perspective.	http://jjwanglab.org/gwas3d	Li et al., 2013
	HumanBase (NetWAS)	HumanBase is the integration of GWAS and tissue-specific networks to identify relevant disease-gene associations. It contains 144 tissue-specific functional networks and 214 biological process-specific functional networks. GWAS data files can be uploaded for analysis or HumanBase predicted tissue-specific interactions can be downloaded.	http://hb.flatironinstitute.org/	Greene et al., 2015
	EnhancerAtlas	EnhancerAtlas provides annotation of enhancers in the human genome and contains enhancers for 76 cell lines and 29 tissues. The database allows users to examine experimental evidences for predicted enhancers in a given genomic region; compare enhancers across different cell/tissue types; identify enhancers associated with a gene; predict genes regulated by a set of *cis*-regulatory elements.	http://enhanceratlas.org/	Gao et al., 2016
	3D Genome Browser	3DGenome is a platform to explore publicly available chromatin interaction data (e.g., Hi-C, ChIA-PET, Capture Hi-C, and PLAC-seq). It also provides multiple methods to link distal *cis*-regulatory elements with their potential target genes.	http://3dgenome.org	Wang et al., unpublished
	4DGenome	4DGenome is a repository for chromatin interaction data (i.e., 3C, 4C, 5C, ChIA-PET, and Hi-C) and bioinformatically predicted interactions (i.e., IM-PET). Records can be queried by genomic regions, gene names, organism, and detection technology.	https://4dgenome.research.chop.edu/	Teng et al., 2015
	3DSNP	3DSNP contains publicly available data from Hi-C experiments and can be queried by variant, gene or genomic region.	www.cbportal.org/3dsnp/index.html	Lu et al., 2017
	Chromatin Chromatin Space Interaction	CCSI presents ∼3,000,000 chromatin interaction pairs with annotation of genes, enhancers and SNPs in many cell lines of human, mouse and yeast. The data was obtained by means of 3C, 4C, 5C, ChIA-PET, and Hi-C technology and can be searched by Ensembl ID, gene name, or chromatin fragment.		Xie et al., 2016
Functional genomic tools	IM-PET	IM-PET integrates gene expression and epigenomic data to predict genes targeted by enhancers.	http://tanlab4generegulation.org/IM-PET.html	He et al., 2014
	PreSTIGE	PreSTIGE makes cell-specific predictions of gene/enhancer pairs by integrating H3K4Me1 histone modification and gene expression data. Publicly available data for 12 cell types can be browsed by gene or enhancer.	http://genetics.case.edu/prestige/	Corradin et al., 2014
Genetic associations	OMIM	OMIM is an online database of >24,000 human genes and all known genetic disorders (including associated variants) that is updated daily and can be queried for clinical features, phenotypes, and genes.	www.omim.org/	Amberger et al., 2015
	GWASdb	GWASdb is a database of published GWAS that can be searched by trait, variant identifier, study or gene. Catalog contains data from 3,092 publications and 53,096 unique SNP-trait associations.	www.ebi.ac.uk/gwas/	MacArthur et al., 2017
	Open Targets	Open Targets collates publicly available data to enable the identification of genes associated with disease through genetic variants and gene expression. Genes can also be queried to identify disease associations.	www.targetvalidation.org/	Koscielny et al., 2017
	Phenome Wide Association Studies	>3,000 GWAS variants were analyzed for associations with 1,358 clinical phenotypes in 13,835 European-ancestry individuals from the Electronic Medical Records and Genomics network. Data can be queried by gene, variant, or clinical phenotype.	https://phewascatalog.org/	Denny et al., 2013

## Identification of Causal Genes by Integrating Genotype and Gene Expression Data

The methods described above are useful in identifying target genes but it is still necessary to demonstrate the effect of trait-associated variants on target gene activity. The directionality of the effect is also crucial information that is used to inform the need for drugs with either antagonistic or agonistic actions on the target. *In vitro* experiments such as reporter gene assays can provide this information by identifying trait-associated variants that modulate the promoter activity of target genes through regulatory elements (Glubb et al., 2015, 2017). A powerful complementary approach is to link the genotype of trait-associated variants to gene expression using *in vivo* data, thus identifying target genes and the directionality of the effect of trait-variants. Expression quantitative trait loci (eQTL) analyses are useful in this regard as they can provide genome-wide lists of genetic variants that associate with gene expression in a particular tissue. There are now a number of eQTL databases (**Table 1**) that can be queried to determine if a trait-associated variant (or variants in linkage disequilibrium) associates with the expression of a specific gene. One of the most comprehensive of these in terms of the diversity of data is the Genotype-Tissue Expression (GTEx) project (Ardlie et al., 2015), which now has eQTL data available for 44 human tissues. Although some tissues currently have a relatively small number of samples (*n* < 100) and consequently suffer from low statistical power for eQTL analysis, data generation is ongoing (Ardlie et al., 2015). Furthermore, the GTEx project provides splicing QTL data, enabling the identification of genetic variants that associate with alternative gene transcripts. Other eQTL studies already include data from a large number of individuals providing statistical power to detect both *cis*- and *trans*-eQTLs with high confidence. For example, Westra et al. (2013) identified eQTLs using data from more than 5,000 blood samples (**Table 1**; Blood eQTL browser).

The linking of gene expression and genotype data can be applied at a multi-variant or gene-based level by combining genotype data to determine the cumulative effect of genetic variants on expression (Gamazon et al., 2015; Gusev et al., 2016). These data are used to predict gene expression levels in cohorts of genotyped individuals, allowing case-control transcriptome-wide association studies to examine whether the predicted gene expression associates with clinical phenotypes and the potentially causal genes identified could provide further targets for drug repositioning.

## Tools for Identification of Drug Repositioning Candidates

Once evidence indicates that a gene is likely regulated by a trait-associated variant, the next step would be to assess whether an existing drug can be repositioned to target this gene or its encoded protein. Numerous databases can be accessed for this purpose, summarized in **Table 2**. These include databases that link drugs to genes through their known pharmacological targets (e.g., DrugBank and ChEMBL). However, many drugs have an array of off-target effects and these unintended pharmacological interactions are often not well known. Therefore, to identify additional pharmacological targets, other databases extract data from the literature that demonstrate the effects of drugs on gene/protein expression and function [e.g., Comparative Toxicogenomics Database (CTD)] or binding interactions between drugs and proteins [e.g., The Binding Database (BindingDB)].

**Table 2:** Databases for the identification of drug candidates for repositioning.

Database	Description	Website	Citation
DGIdb	DGIdb contains 28,000 drug-gene interactions from 27 sources and can be queried by gene or drug.	http://dgidb.genome.wustl.edu/	Wagner et al., 2016
RE:fine Drugs	RE:fine provides a triad approach linking drugs to genes and disease at the same time as linking genes/variants to disease. The database contains 1,770 diseases, 916 drugs, and 567 genes and findings can be queried by drug, disease, or gene.	http://drug-repurposing.nationwidechildrens.org/	Moosavinasab et al., 2016
CMap	CMap is a library of nearly 500,000 gene expression signatures from human cell lines exposed chemical and genetic perturbation, and supports queries by gene and drug. The database also contains detailed drug information including known targets, stage of development and mechanism of action.	https://clue.io/	Lamb et al., 2006
DrugBank	DrugBank contains information on 9,591 FDA approved and experimental drugs with links to 4,661 targets. DrugBank supports general queries by drug, target, pathway and indication.	https://www.drugbank.ca/	Law et al., 2014
ChEMBL	ChEMBL contains 11,538 target and 2.1 million compound records that can be searched by ligand or target and browsed for drugs, targets or indications.	https://www.ebi.ac.uk/chembl/	Gaulton et al., 2017
PharmGKB	PharmGKB contains information on 621 drugs, 118 pathways and 474 pharmacogenetic labels as well as encompassing clinical information including gene-drug associations and genotype–phenotype relationships. PharmGKB can be queried for molecule, gene, variant, or combination.	https://www.pharmgkb.org/	Whirl-Carrillo et al., 2012
CTD	The Comparative Toxicogenomics Database (CTD) contains 34 million relationships between drugs (and other chemical entities) and genes/proteins. and can be queried by chemical, chemical-gene interaction, gene, gene form, disease, pathway, organism, or gene ontology.	http://ctdbase.org/	Davis et al., 2017
STITCH	STITCH is a database of known and predicted interactions between chemical entities and proteins, containing data from ∼500,000 chemicals, 9.6 million proteins and 1.6 billion interactions. The database supports queries by chemical, protein or gene name as well as by chemical structures or protein sequences.	http://stitch.embl.de/	Szklarczyk et al., 2017
BindingDB	BindingDB is a database of measured binding affinities focusing on the interactions of proteins considered to be drug-targets and small, drug-like molecules. It contains data for 7,225 protein targets and 621,060 small molecules. Searches can be conducted by small molecule, target, or assay.	http://bindingdb.org/bind/index.jsp	Gilson et al., 2016

A novel approach for identifying drug repositioning opportunities is provided through the Connectivity Map (CMap) database (**Table 2**). CMap is a resource that uses gene expression changes in response to drug treatment and gene perturbation (i.e., knockdown/overexpression) to find relationships between genes and drugs. CMap contains over one million gene expression signatures from the treatment of a variety of cell types with drug and gene perturbations. Differential expression signatures that arise from treatment can be compared to signatures in the database to perform both positive and negative correlations. These data could be applied to the identification of drug candidates for repositioning. For example, if a gene is known to be down-regulated by a trait-associated variant, a search can be performed to identify drugs that may have a beneficial expression signature, i.e., drugs with a similar signature to the opposite gene perturbation (in this case gene overexpression).

## Pre-Clinical Validation of Drug Repositioning

Before clinical drug repositioning trials can be performed, pre-clinical studies are crucial to validate targeting of the gene or protein of interest and demonstrate a desired effect in cellular or animal models. Definitive proof that the target is necessary for the desired effect is not a trivial exercise and requires manipulation of the target in the model system, which is often accompanied with caveats (Kaelin, 2017). With the vast array of tools now available, it is now relatively straightforward to genetically manipulate targets by transcript overexpression/knockdown (e.g., cDNA clones and siRNA), gene knockout/knock-in and even by the introduction of gain or loss of function mutations (e.g., CRISPR/Cas9). These gene perturbation techniques can also be applied in a high throughput fashion to cellular or animal models using pooled cDNA, siRNA, or gRNA libraries to characterize gene function (Joung et al., 2017; Tsherniak et al., 2017) with image-based profiling providing the capability to measure multiple phenotypes at the same time (Caicedo et al., 2016). However, gene perturbation approaches themselves often have off-target effects that might confound findings and thus experiments need to be well controlled to ensure the correct interpretations are made (Kaelin, 2017). To unambiguously validate targets, rescue experiments are required. In these experiments, the desired phenotype is reversed (or rescued) by a drug resistant version of the target or reintroduction of the target through some means which is resistant to the original gene perturbation or ablation (Kaelin, 2017). Difficulties in reproducing experimental results between laboratories (Prinz et al., 2011) also highlights the need for multiple experimental lines of evidence and findings that are robust to different conditions and models.

## The Identification of Further Diseases for Repositioning

Another method for identification of drug repositioning opportunities leverages the fact that genetic variation can have pleiotropic effects and associate with multiple clinical phenotypes. Therefore, a drug successfully repositioned using genetic data may be able to be repositioned for the treatment of further diseases if the underlying genetic variant(s) has a pleiotropic effect. A relatively new technique that can be applied to this is the phenome-wide association study (PheWAS), where a single variant is tested for association with a large number of phenotypes, enabling the identification of variants that confer susceptibility to multiple diseases (Denny et al., 2010). Databases that contain de-identified Electronic Medical Records (EMRs) are an efficient source of data for PheWAS (Manolio et al., 2009). EMR databases contain longitudinal health records that include prescription records, family histories, laboratory and image testing results, physician notes and, importantly, the International Classification of Disease codes (Hebbring, 2014). An additional approach would be to use genetic correlation analyses, such as LD-score regression, that use GWAS data to identify genetic similarities between diseases which could then provide an avenue for further repositioning.

## Limitations of Drug Repositioning and Future Directions

Although drug repositioning appears to have many advantages over traditional drug development, there are some caveats. Firstly, there needs to be a drug that can be repositioned against the target of interest. This may not always be the case and, therefore, drug repositioning should be considered a complementary approach to the development of novel drugs. In terms of clinical trials, Phase I studies may still be necessary if an increased dosage of the repositioned drug is required, if a new drug delivery method is used, or if it the drug is intended to be used in a new population. Nevertheless, by repositioning a drug against a target on the basis of genetic evidence, the increased likelihood of approval may still offset the costs of Phase I trials. Intellectual property for drug repositioning needs to be considered as drug repositioning uses drugs that are already published cannot be patented because they have already been publicly disclosed. Lack of patentability reduces opportunity for profit dis-incentivizing pharmaceutical companies from pursuing that target. For example, generic drugs with well characterized safety profiles may appear amenable to drug repositioning, but a lack of intellectual property could prevent pharmaceutical companies from recouping costs spent on testing in clinical trials (Nosengo, 2016). However, repositioning drug candidates could be refined or modified to provide better targeting and thus generate new intellectual property. Current patent law regarding drug repositioning is complex and inconsistent and thus greater clarity and uniformity is required (Kremer and Jones, 2015). It is also important that exclusivity and patent strategies exist to provide incentives for pharmaceutical companies to invest in this area of research (Kremer and Jones, 2015). Furthermore, the drug repositioning process could be promoted by collaborative models involving academic researchers, pharmaceutical companies and other stake holders. For example, the MRC-Industry Asset Sharing Initiative^1^ and the NIH National Center for Advancing Translational Sciences (NIH-NCATS^2^) aim to deliver treatments and cures for disease to patients faster by improving the translational process.

## Conclusion

Drug repositioning potentially provides a faster and cheaper approach to the development of new therapies and, if targets have a genetic basis, should carry less risk. Yet, a concerted effort still needs to be made to overcome the bottleneck of identifying targets from large-scale genetic studies and rigorous approaches need to be taken in the pre-clinical validation of drug repositioning to maximize likelihood of success in clinical studies.

## Author Contributions

Conception or design of the work; the acquisition, analysis and interpretation of data for the work (J-LP, DG, and TO). Drafting the work or revising it critically for important intellectual content (J-LP, DG, and TO). Final approval of the version to be published (J-LP, DG, and TO). Agreement to be accountable for all aspects of the work in ensuring that questions related to the accuracy or integrity of any part of the work are appropriately investigated and resolved (J-LP, DG, and TO).

## Conflict of Interest Statement

The authors declare that the research was conducted in the absence of any commercial or financial relationships that could be construed as a potential conflict of interest. The reviewer SP and handling Editor declared their shared affiliation.

## Footnotes

1 https://www.mrc.ac.uk/funding/browse/industry-asset-sharing-initiative/mrc-industry-asset-sharing-initiative/

2 https://ncats.nih.gov/
